# Robust nanobubble and nanodroplet segmentation in atomic force microscope images using the spherical Hough transform

**DOI:** 10.3762/bjnano.8.257

**Published:** 2017-12-01

**Authors:** Yuliang Wang, Tongda Lu, Xiaolai Li, Shuai Ren, Shusheng Bi

**Affiliations:** 1 School of Mechanical Engineering and Automation, Beihang University, Beijing 100191, P. R. China

**Keywords:** atomic force microscopy, Hough transform, morphological characterization, nanobubbles, nanodroplets, segmentation

## Abstract

Interfacial nanobubbles (NBs) and nanodroplets (NDs) have been attracting increasing attention due to their potential for numerous applications. As a result, the automated segmentation and morphological characterization of NBs and NDs in atomic force microscope (AFM) images is highly awaited. The current segmentation methods suffer from the uneven background in AFM images due to thermal drift and hysteresis of AFM scanners. In this study, a two-step approach was proposed to segment NBs and NDs in AFM images in an automated manner. The spherical Hough transform (SHT) and a boundary optimization operation were combined to achieve robust segmentation. The SHT was first used to preliminarily detect NBs and NDs. After that, the so-called contour expansion operation was applied to achieve optimized boundaries. The principle and the detailed procedure of the proposed method were presented, followed by the demonstration of the automated segmentation and morphological characterization. The result shows that the proposed method gives an improved segmentation result compared with the thresholding and circle Hough transform method. Moreover, the proposed method shows strong robustness of segmentation in AFM images with an uneven background.

## Introduction

In the past two decades, interfacial nanobubbles (NBs) [1–3] and nanodroplets (NDs) [4–6] have been attracting more and more attention because of their enormous potential in numerous applications. It is reported that NBs can ameliorate oxygen mass transfer to living microorganisms [7], reduce drag force at solid–liquid interfaces in micro/nanofluidics [2,8–9], and enhance ultrasonic tumor imaging contrast [10]. Regarding NDs, they can be applied to fabricate nanolenses on solid surfaces to modify them for antireflection and light harvesting [11], adsorb onto nanocrystal and microcrystal surfaces for direct heterogeneous engineering [12], and help functional oil deposition from emulsions [13]. NBs and NDs can also act as templates for nanomaterial engineering, such as assembling nanoparticles into nanorings [14], generating nanostructures on polymer [15] and highly oriented pyrolytic graphite (HOPG) surfaces [16].

In general, NBs and NDs are 100–800 nm in width and 10–100 nm in height. They are generally studied by atomic force microscopes (AFM) due to their high spatial measurement resolution. The morphological characterization of NBs and NDs, such as contact angle, size, density, and volume, is generally required in their studies. For example, in the study of wettability properties of NDs, contact angle is generally applied [17–18]. In the study of influence factors for NB/ND formation, such as temperature [19–21] and gas type [22–23], their size and density are statistically analyzed.

Segmentation of NBs and NDs is a primary operation to characterize them in AFM images. Normally there are several tens or even hundreds of them in one AFM image. The manual segmentation is tedious and time consuming. As a result, the automated segmentation methods become necessary. Technically speaking, NB/ND segmentation includes two aspects, NB/ND localization and boundary detection. The NB/ND localization is a process of determining their location in AFM images, while boundary detection is a process of extracting contours which are as close as possible to their actual boundaries. Through segmentation, their size, density, contact angle and even volume can be extracted.

**Figure 1:**
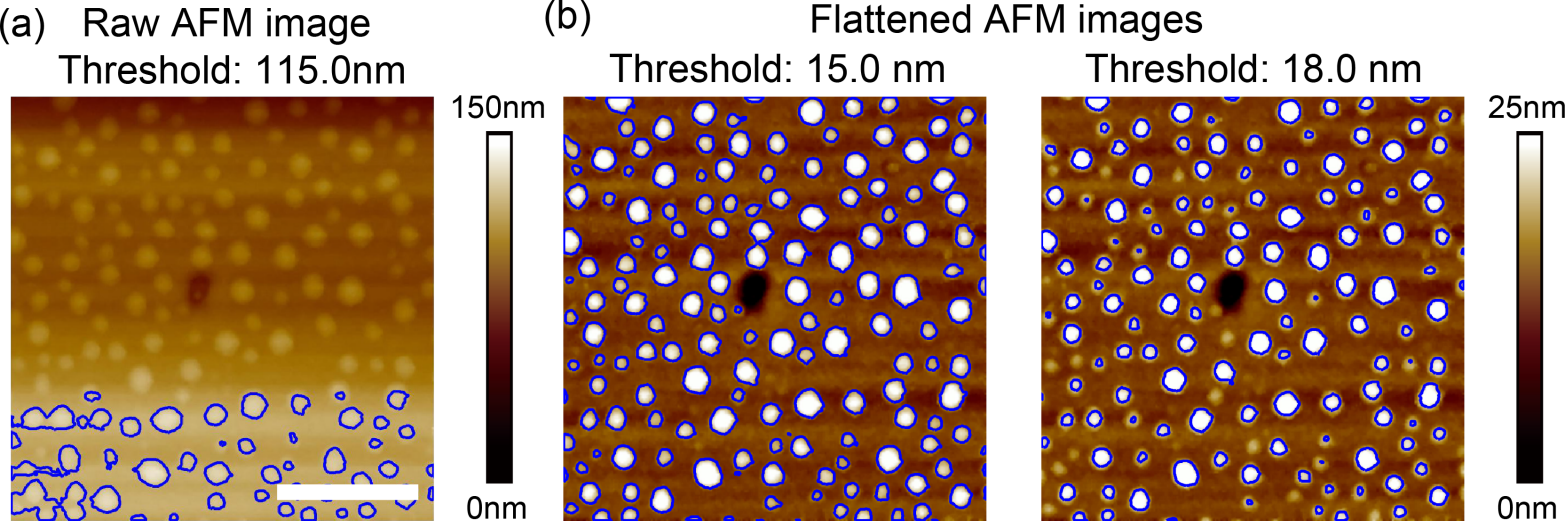
Segmentation of nanobubble AFM images using the thresholding method. (a) In a raw AFM image with a rough background, only part of the nanobubbles can be detected. (b) After flattening, nearly all nanobubbles are detected when a proper threshold value is selected (left). However, the segmentation is sensitive to the threshold value. A relatively higher threshold value could cause under-segmentation (right). The scale bar is 500 nm.

The major difficulty in automated NB/ND segmentation is the uneven background of AFM images, either because of the thermal drift and hysteresis of AFM scanners, noise [24–25] or the actual topography of the sample surfaces (e.g., HOPG). In general, it is difficult to establish one source of the uneven background from the others in AFM images. Practically, researchers use a plane fitting method to aggressively flatten AFM images to improve the contrast for smaller objects by assuming sample surfaces are actually flat, which is not always true. Figure 1a is a raw AFM image of NBs on a polystyrene (PS) surface. One can see that the image height of the background increases along the *y* direction. This is mostly due to thermal drift of the AFM scanner. By applying image flattening, an AFM image with improved contrast can be obtained, as shown in Figure 1b.

Today, the most widely applied segmentation method is the thresholding method [19,26] in which the choice of threshold value is a matter of great concern due to the uneven background of AFM images. A too small threshold value will cause over-segmentation, while a too large one will cause under-segmentation and thus the under-estimation of the NB/ND height and volume. For the image shown in Figure 1a, the segmentation result using the thresholding method is unsatisfactory. Only NBs in the higher region are detected, while the rest are all ignored. For these kind of images, they must be flattened to get improved segmentation result in the thresholding method (Figure 1b). For the flattened images, if proper threshold values are applied, they can be correctly detected, as shown in the left figure in Figure 1b. However, the thresholding method is sensitive to the selection of a threshold value. As shown in the right figure in Figure 1b, a relatively larger value causes under-segmentation. Moreover, even for the segmentation result shown in the left figure of Figure 1b, the detected contours for individual NBs are not converged to their actual boundaries, as we previously reported [27].

In addition to the over- or under-segmentation, another challenge in NB/ND segmentation is the boundary detection. In the thresholding method, a threshold value is selected and only the portion higher than the value can be taken as NBs and NDs. Due to the existence of thermal drift, noise and hysteresis of AFM scanners [25], their vertical position in an AFM image may vary greatly. It is a difficult challenge to determine the threshold value in the thresholding method, since they do not share the same footprint height, even for the adaptive thresholding method. To solve the problem, we have proposed a contour expansion method [27]. In this method, AFM images were first preliminarily detected using the thresholding method. The active contour method is then applied to the boundaries of the preliminarily detected NBs to achieve the optimized boundary detection.

Interfacial NBs and NDs are all spherical-cap-shaped objects in AFM images. Based on this, some efforts have been have been focused on shape-based image segmentation. Tan et al. applied the circle Hough transform (CHT) to implement segmentation of micro-spheres in optical images [28]. Since the CHT method only utilizes boundary information, it can achieve a good segmentation for micrometer-sized spheres with enough data points. Regarding NBs and NDs, we find that it is difficult to get an optimized segmentation result with this method because of the limited number of data points and noisy images.

In this study, we proposed a two-step method for NB/ND segmentation in AFM images. In the first step, we applied a spherical Hough transform (SHT) to locate them in AFM images. Based on the SHT result, the initial contours of NBs and NDs were extracted. Then, the contour expansion method [27] was applied to the initial contours to get the optimized boundary detection based on the active contour model [29], followed by the morphological characterization.

## Experimental

### Imaging of nanobubbles and nanodroplets

In this study, NBs were produced on a PS surface, which was spin-coated as a thin film on a silicon (100) substrate at a speed of 500 rpm. The silicon substrate was cleaned in a sonic bath of piranha, acetone and then water for 30 mins before spin coating. PS particles (molecular weight 350,000, Sigma-Aldrich) were dissolved in toluene (Mallinckrodt Chemical) to obtain the solution for spin coating. Upon the immersion of the PS film in deionized (DI) water, NBs are spontaneously generated on the PS surface.

NDs were obtained on a freshly cleaved HOPG surface. To form the NDs, 10 µL of poly(dimethyl siloxane) (PDMS) solution was dissolved in 20 mL of chloroform solution. The obtained solution was then deposited on the surface to generate a thin liquid film. After the volatilization of the solution, the surface was immersed in DI water. The PDMS NDs were then obtained on the surface.

In this study, the PS and HOPG surfaces were scanned in both air and DI water using a commercial AFM (Resolve, Bruker) in tapping mode with 96% setpoint value. Silicon cantilevers (NSC36/ALBS, MikroMasch) with a quoted stiffness of 0.6 N/m and tip radius of 8 nm were used for scanning. The measured resonance frequencies of the cantilever were 55 kHz and 16 kHz in air and in DI water, respectively. The scanning frequency was 2 Hz and the scanning angle was 0°.

**Figure 2:**
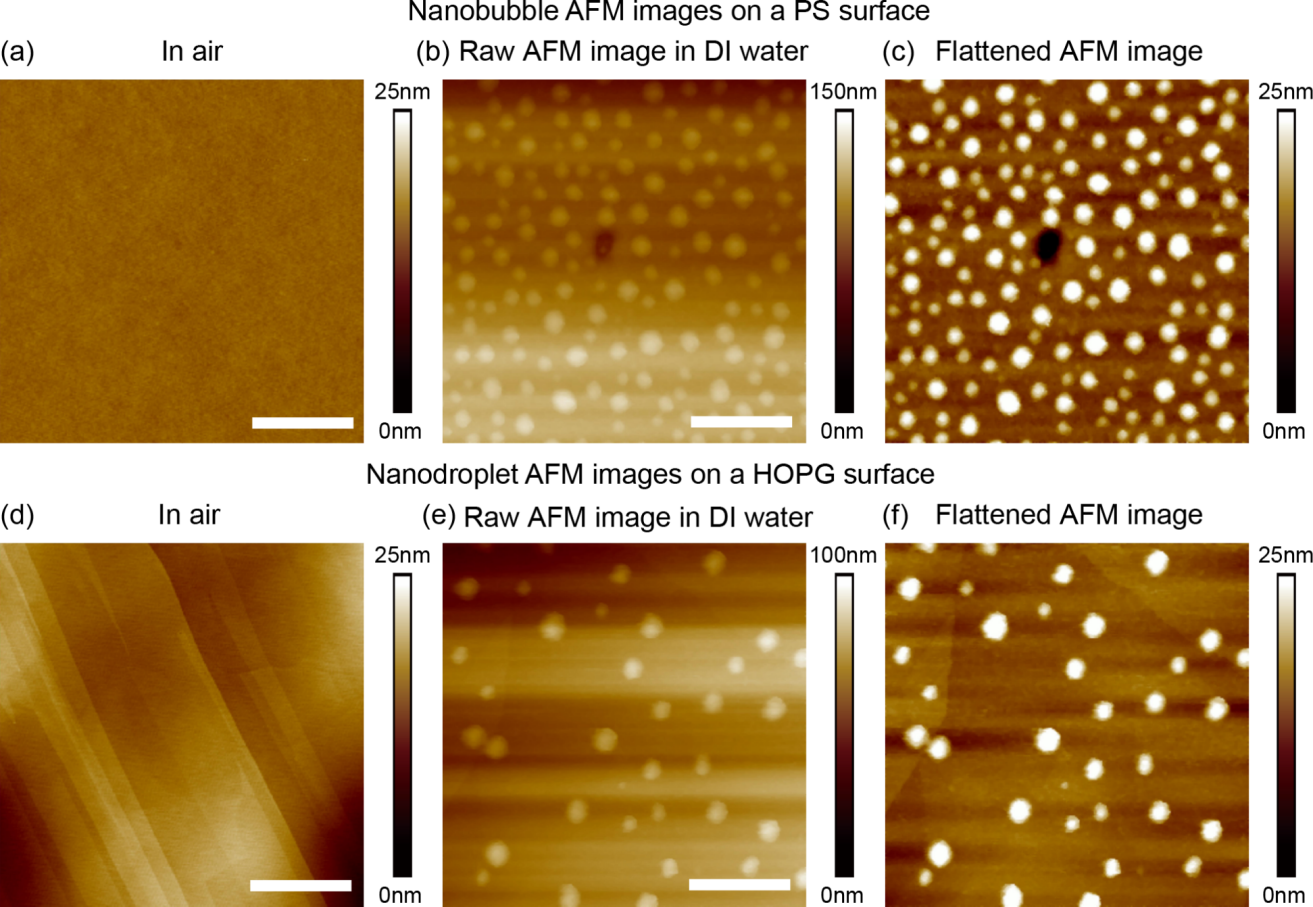
AFM height images of nanobubbles and nanodroplets. (a) and (d) are the polystyrene and HOPG surfaces in air. (b) and (e) are the raw AFM height images of nanobubbles and nanodroplets in DI water. (c) and (f) are flattened AFM height images of nanobubbles and nanodroplets in DI water. The scale bar is 500 nm.

Figure 2a and Figure 2d are the AFM height images in air for the PS and HOPG surfaces, respectively. After both samples were immersed in DI water, the NBs and NDs were obtained on the surfaces. The raw AFM images for the NBs and NDs are shown in Figure 2b and Figure 2d, respectively. One can see that both images have an uneven background, which is believed to be due to the thermal drift. The flattened images are shown in Figure 2c and Figure 2f, respectively.

### Characterization of nanobubbles and nanodroplets

**Figure 3:**
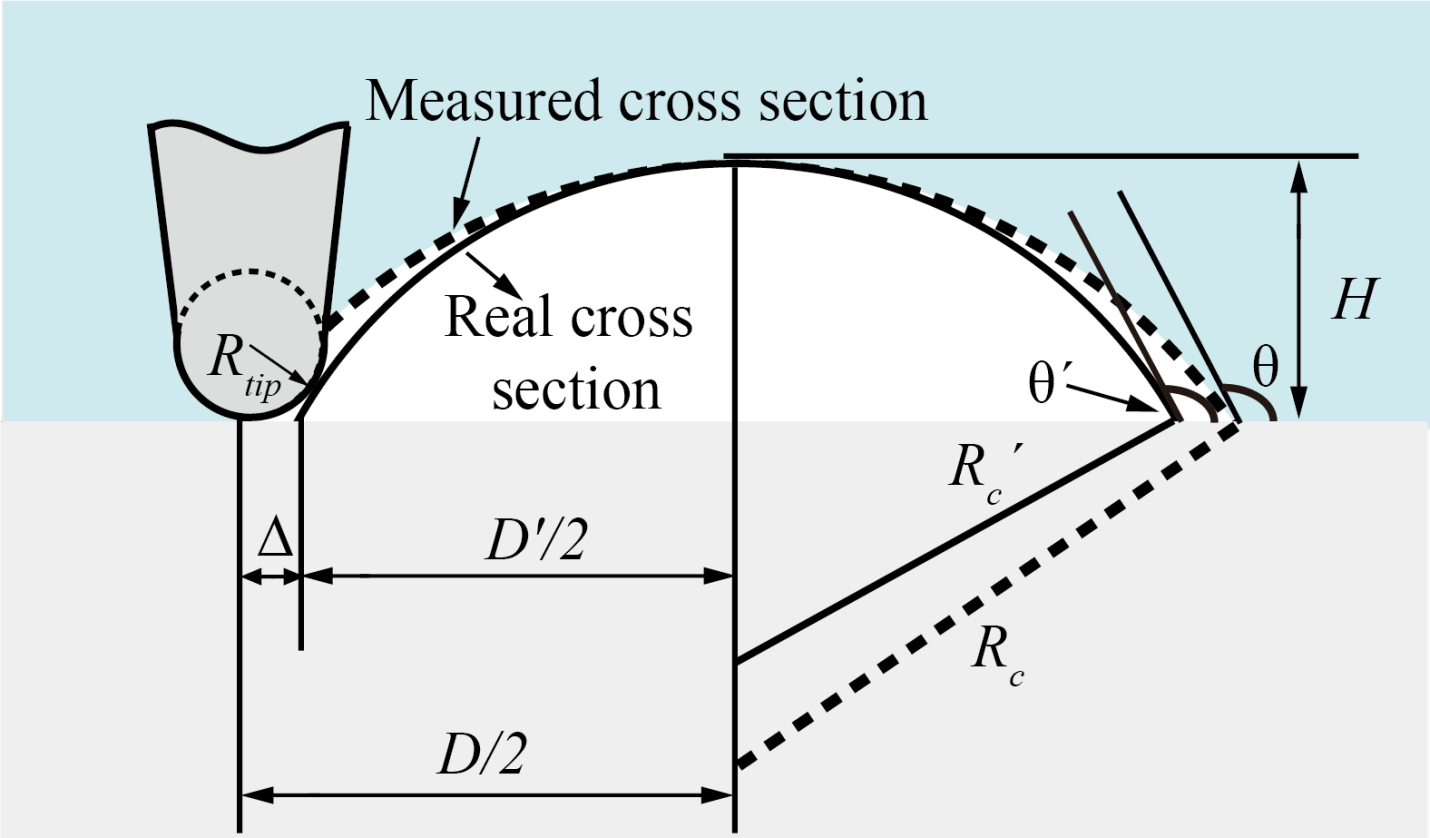
Sketch of the morphological characterization and AFM tip correction for nanobubbles and nanodroplets. The solid line indicates the real cross section, while the dotted line indicates the measured cross section.

Both NBs and NDs have a spherical-cap shape. To facilitate the morphological characterization, some parameters are introduced in this paper, as shown in Figure 3. In the figure, *H* refers to the height of a NB or ND, *D* and θ are the width and contact angle, respectively. After segmentation, *H* and *D* can be directly obtained and the contact angle can then be calculated.

It is known that the topography image obtained from an AFM image is the convolution of the AFM tip and substrate morphologies [30–32]. In the case of the spherical-cap-shaped NBs and NDs, the influence of the AFM tip on the contact angle measurement is illustrated in Figure 3. From the figure, one can see that the tip radius results in the overestimation of the width *D*. The deconvolution of the AFM tip gives a corrected expression of radius *R*_c_′, width *D*′ and contact angle θ′, as

[1]
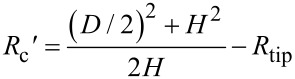


[2]
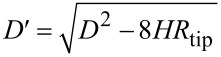


[3]
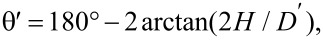


where *R*_tip_ is the radius of AFM tip.

### Algorithms of nanobubble and nanodroplet image segmentation

**Figure 4:**
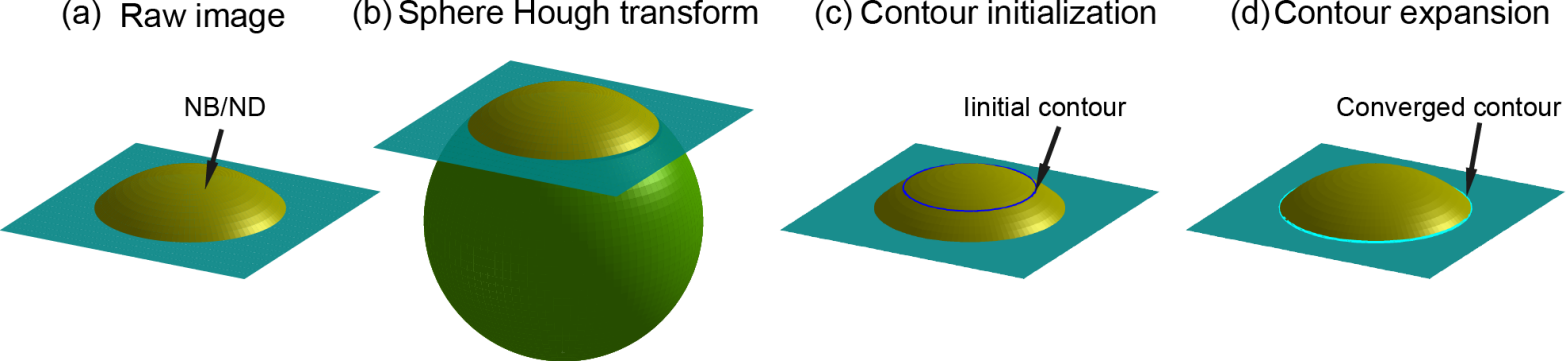
Illustration of the proposed two-step nanobubble/nanodroplet (NB/ND) segmentation based on the spherical Hough transform combined with the contour expansion operation. (a) Sketch of a simulated AFM image. (b) The spherical Hough transform is applied to preliminarily detect the NBs/NDs, where the sphere represents the highest possibility which NB/ND caps belongs to. (c) The contour of the preliminarily detected NB/ND is extracted and taken as the initial contour for the NB/ND. (d) Detection of the optimized boundary with the contour expansion method.

The principle of the proposed two-step segmentation method is illustrated in Figure 4. A simulated NB/ND image shown in Figure 4a is first converted into a 3D point cloud. The SHT is then applied to the point cloud to preliminarily determine the position and size of the NBs/NDs (Figure 4b), based on which the initial contour of the NBs/NDs can be extracted (Figure 4c). To get the optimized boundary, the contour expansion method [27] will be applied to the initial contour. Driven by the field of gradient of the AFM image, the initial contour will be converged towards the NB/ND boundary and the final contour can then be obtained (Figure 4d).

### The spherical Hough transform

**Figure 5:**
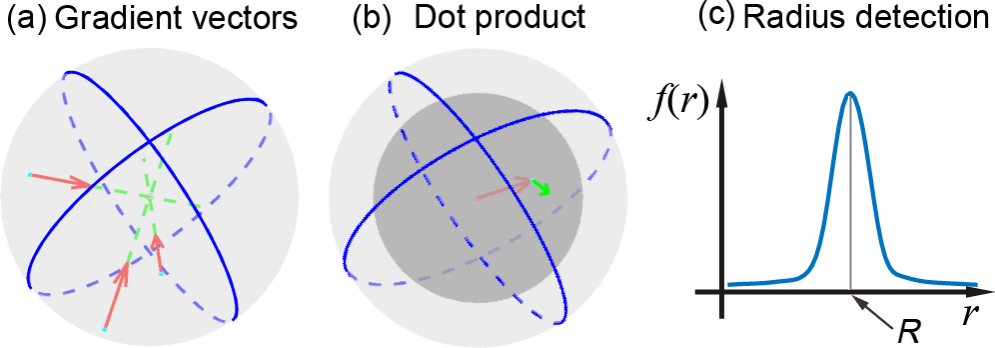
Schematic diagram showing the principle of the spherical Hough transform. (a) Calculation of gradient vectors for all data points in a 3D image. The center of the sphere is detected by searching for a point which is crossed by most gradient vectors. (b) The sum of the dot product is used to determine the radius of the sphere. For a point with the distance *r* to the sphere center, the dot product of the kernel vector (red arrow) and the gradient vector of the point (green arrow) is first calculated. The sum of all these dot products is defined as *f*(*r*). (c) The value *r* corresponding to the maximum value *f*(*r*) is taken as the radius *R* of the sphere.

The SHT is generally used to detect objects with spherical shapes in 3D images or point clouds [33–34]. In the SHT, a parameter space has the same dimension as AFM images. The principle of the method is shown in Figure 5. In Figure 5a, gradient vectors for data points in an input image are first calculated. A point in parameter space gets a vote when the point at the same position in the image space is crossed by a vector line. According to the fact that all gradient vectors point to the center, a local maximum will be obtained around the center area in the parameter space.

After the sphere centers are detected, the next step is to determine the sphere radii. A sum of the dot product *f*(*r*) at a given distance *r* to the detected sphere center is proposed as [35–36]:

[4]
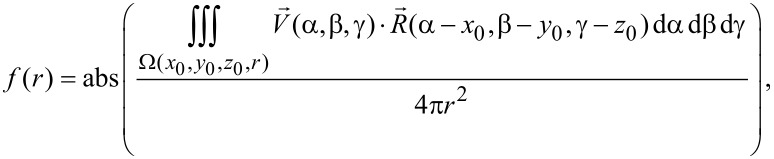


where

[5]
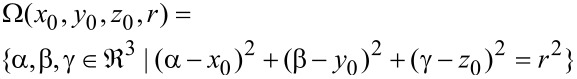


[6]
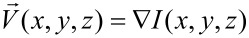


[7]
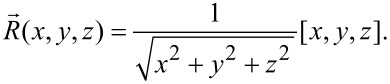


For each point with a given distance *r* to a detected sphere center, the dot product of the kernel vector (Equation 7, and red arrow in Figure 5b) and gradient vector of the point (Equation 6, and green arrow in Figure 5b) indicates how possible the point belongs to the sphere. By summing up the dot product for all the data points with the same distance *r* to the center, *f*(*r*) will be obtained. For *r* in a specific detection range, the corresponding *f*(*r*) can then be obtained (Figure 5c). *f*(*r*) achieves its maximum value when *r* equals to the radius of the sphere.

**Figure 6:**
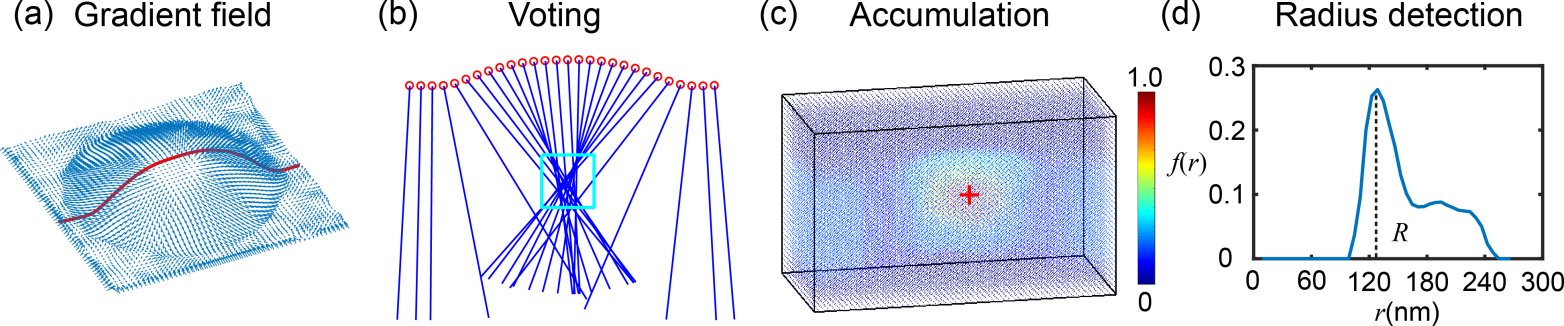
Demonstration of the spherical Hough transform detection of an nanobubble. (a) Gradient vector calculation over a nanobubble surface. (b) Gradient vectors along a cross section indicated in (a). Most of the gradient vectors appear in a small region. (c) Accumulation in the parameter space indicating the frequency of each cell being passed by gradient vectors. The region marked by a red cross is considered as the sphere center of the nanobubble. (d) The change of the sum of the dot product *f*(*r*) along the radial direction from the detected sphere center. It reaches its maximum value at the distance *R*, which is the detected nanobubble radius. After the above operation, the center and radius of the detected sphere can be determined.

In this study, the SHT is used to detect NBs and NDs. In Figure 6a, a raw NB height image is first converted to a point cloud. The gradient vectors can then be calculated. The gradient vectors along a cross section are shown in Figure 6b. One can see that the gradient vectors of the data points go across a small region, which is the region where the NB center is. For each unit cell in the parameter space, the frequency of being crossed by the gradient vectors will be obtained after all the radius vectors are calculated, as shown in Figure 6c. In the figure, the central red region has the highest density and corresponds to the region of the sphere center. After the center is detected, *f*(*r*) can then be obtained, as shown in Figure 6d. In the figure, *R* is the detected radius.

### Optimized boundary detection

**Figure 7:**
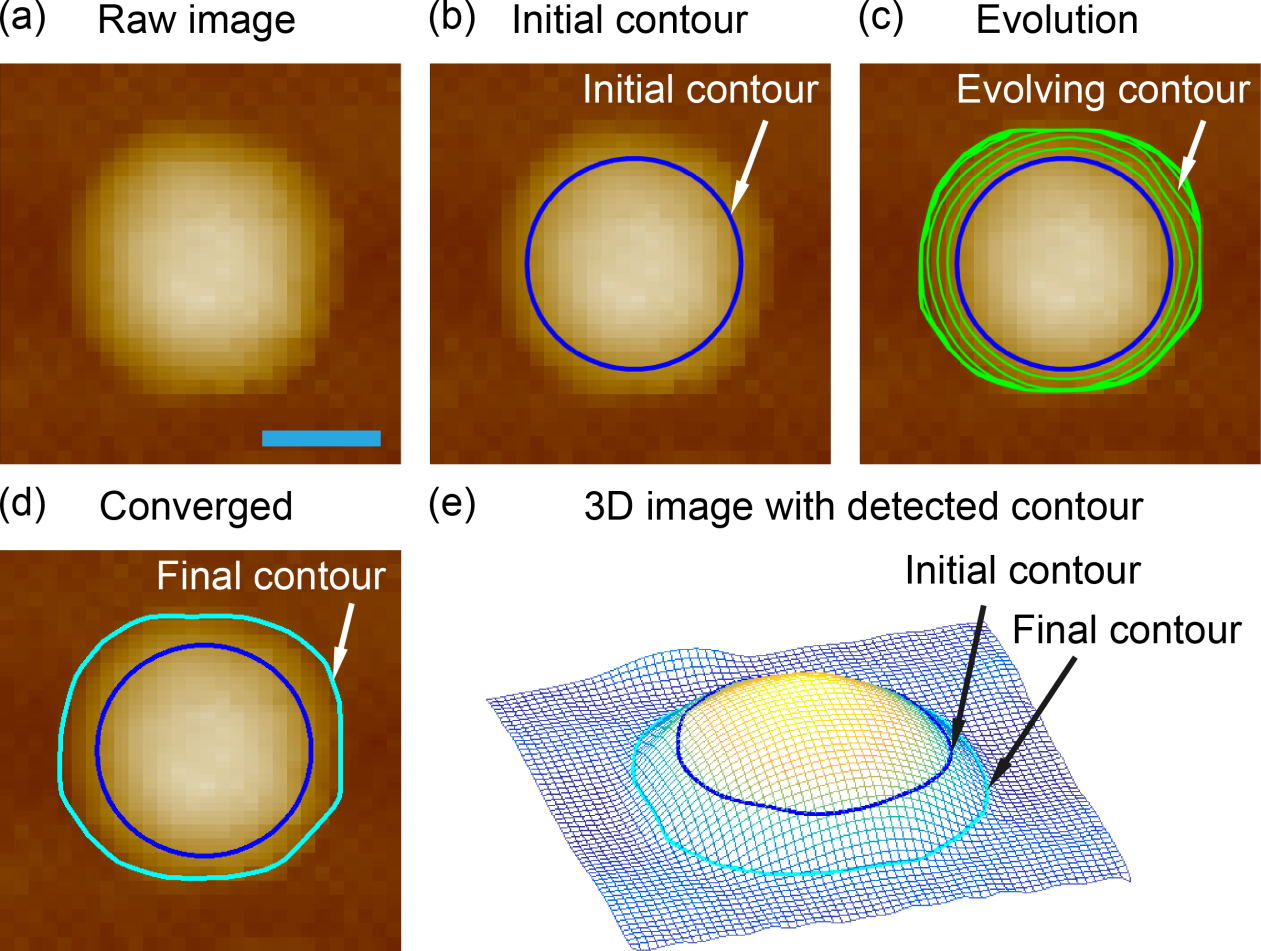
Demonstration of contour expansion operation to obtain the optimized boundary detection for a nanobubble. (a) The raw AFM image of a nanobubble. (b) Extraction of the initial contour after the spherical Hough transform detection of the nanobubble. A circle determined by the detected sphere through the spherical Hough transform is taken as the initial contour for contour expansion. (c) Evolving contours converge towards the bubble boundary driven by the field of gradient in the image. (d, e) The comparison of the initial and converged contours in 2D (d) and 3D (e) mesh plots of the nanobubble. The result shows that the proposed method can achieve a good estimation of the NB/ND boundaries.

To obtain the optimized NB/ND boundaries, the contour expansion operation [27] was applied to the initial contours detected by the SHT using an active contour model. The process is demonstrated in Figure 7. For a NB image shown in Figure 7a, the SHT was first applied. As mentioned earlier, a sphere will be detected for each NB/ND. A circle which has the same *x*, *y* coordinates with that of the detected sphere was taken as the initial contour (blue circle in Figure 7b).

In the contour expansion operation, the initial contours of NBs and NDs are expressed as parameterized curves *v*(*s*) = (*x*(*s*), *y*(*s*)) and their energy function is defined as [29]:

[8]
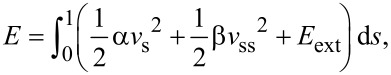


where *v*_s_ and *v*_ss_ are the first and second order partial derivatives of the contour curves, respectively, α and β are two scalar coefficients. The first two items of the energy function represent the internal energy of the contours, which only dependend on the curve geometry. The third item represents the external energy (this is the height value of the AFM images) along the contours. The internal energy enforces the smoothness and continuity of the evolving contours, while the external energy makes contours converge to the region with a lower height value to minimize the total energy.

In practice, the Euler–Lagrange function is usually used to find the minimum of the energy function [29]:

[9]
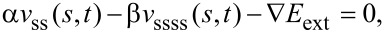


where *v*_ssss_ is the fourth order partial derivative of the contours. For an initial contour shown in Figure 7b, the Euler–Lagrange function can be iteratively solved. Under the impact of both internal and external energy, the contour gradually converges and eventually stops at the NB’s boundary, where the minimum total energy is achieved (green contours in Figure 7c). The converged contour is shown in Figure 7d. Figure 7e shows the mesh plot of the selected NB with both initial and final contours obtained by the contour expansion operation. It is obvious that the final contour gives a good estimation of the boundary.

## Results and Discussion

In this section, a comparison of NB/ND segmentation with three different methods was first conducted. The robustness of the proposed method in processing the raw and flatten images is then verified, followed by the morphological characterization.

### Comparison of three methods

**Figure 8:**
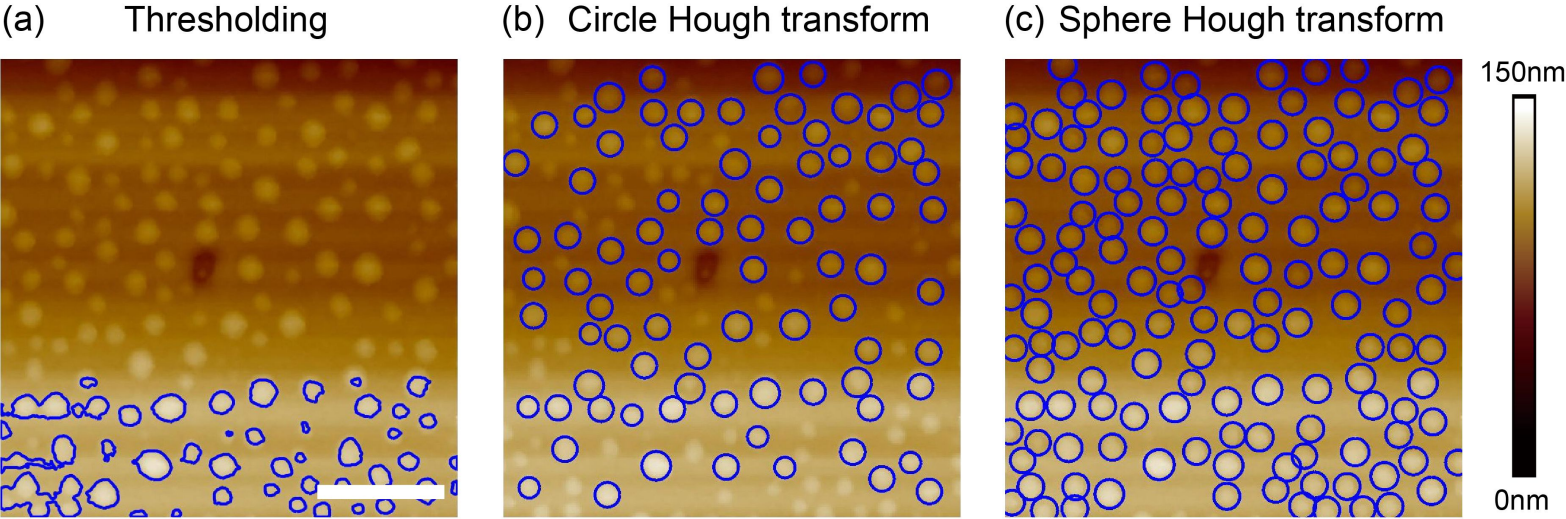
Comparison of nanobubble detection in a raw AFM image with the (a) thresholding method, (b) circle Hough transform, and (c) spherical Hough transform. The thresholding method is sensitive to the uneven background and can only partially detect the bubbles in the region with larger height. The circle Hough transform can only detect larger nanobubbles, while the spherical Hough transform can detect all the nanobubbles in the image. The scale bar is 500 nm.

To validate the SHT in the preliminary segmentation of NBs and NDs, a comparison of segmentation methods for a raw AFM image (without flattening) was conducted with thresholding, CHT and SHT methods, as shown in Figure 8. As mentioned earlier, the raw AFM image has an uneven background. Figure 8a shows the result obtained with the thresholding method. Since the thresholding method is sensitive to the uneven background, only NBs in the higher region can be segmented.

In Figure 8b, the CHT was applied, which works with the similar principle as the SHT. However, due to the scanning error and noise, the boundaries of NBs and NDs in AFM images are not standard circles in shape. Moreover, for each nanobubble, only dozens of points on the boundaries are used to determine centers. As a result, the CHT could only detect the those with relatively large size and good circular shape, but fails to detect small, unsharp ones. Figure 8c shows the result with the SHT. The SHT utilizes all data points on NB/ND surfaces, which is much more than in the CHT. As a result, the SHT provides a much better detection result, where all NBs in the image can be detected, while only about 60% of the NBs are detected by the CHT.

### Robustness of the proposed method

**Figure 9:**
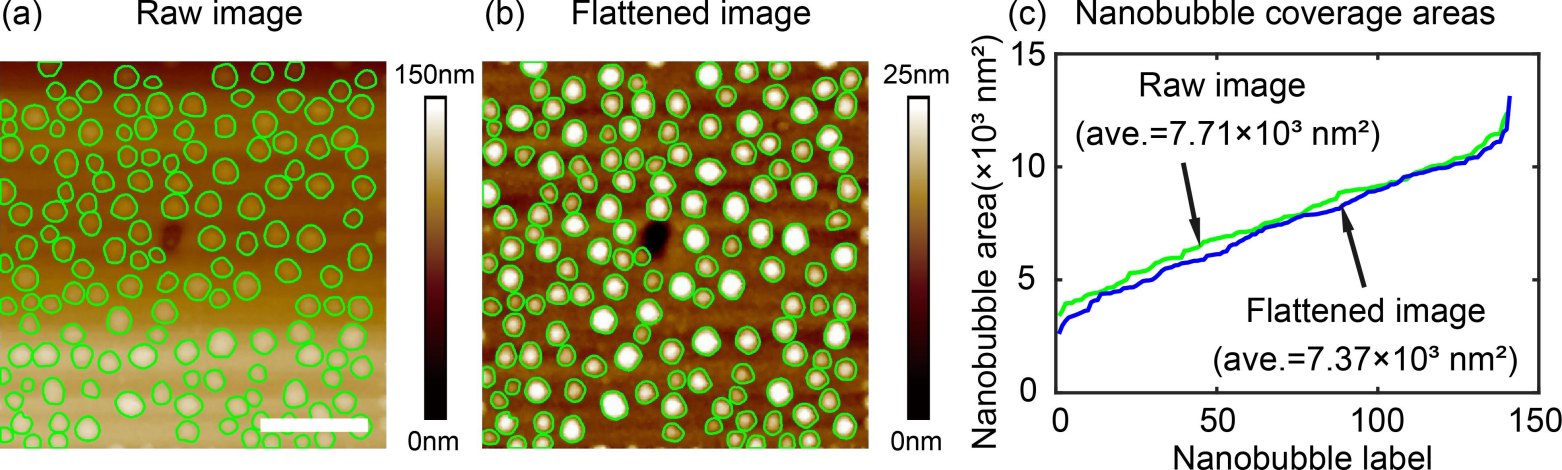
Robustness of the proposed method in nanobubble image segmentation with uneven background. Nanobubble image segmentation in the raw nanobubble image with an uneven background (a) and the flattened image (b) using the proposed method. (c) Comparison of nanobubble coverage areas detected in the raw image and the flattened image using the proposed method. The two results are close to each other, which implies that the proposed method is robust to the uneven background. The scale bar is 500 nm.

By principle, the proposed method is supposed to be robust for NB/ND detection, regardless of whether the AFM images are flattened or not. To verify this, a comparison was conducted with both the raw and flattened AFM images. The SHT and contour expansion steps were sequentially applied to both images by applying the same parameters during segmentation. The segmentation result is shown in Figure 9a and Figure 9b for the raw and flattened AFM images, respectively. In both images, the green contours correspond to detected boundaries. One can see that no matter how the background changes, the proposed method was able to achieve a similar result for both images.

The robustness of the proposed method is further confirmed through a comparison of coverage areas for all detected NBs in the two images, as shown in Figure 9c. In the figure, they are labeled by the ascending order of the areas. The green curve shows the NB coverage areas in the raw AFM image, while the blue curve shows this in the flattened image. It is obvious that the proposed method gives very close results for both images. The average coverage areas for the raw and flattened AFM images are 7.71 × 10^3^ nm^2^ and 7.37 × 10^3^ nm^2^, respectively, which corresponds to a 4.4% difference.

The difference is mostly because the flattening process unavoidably changes the height distribution of NBs. The converged contours change accordingly, leading to the changed coverage areas between the two images. Considering this influence and the small difference, one can conclude that the proposed method is robust to a typical AFM image background. This is especially important for some AFM images whose actual backgrounds are not flattened.

### Morphological characterization of nanobubbles and nanodroplets

**Figure 10:**
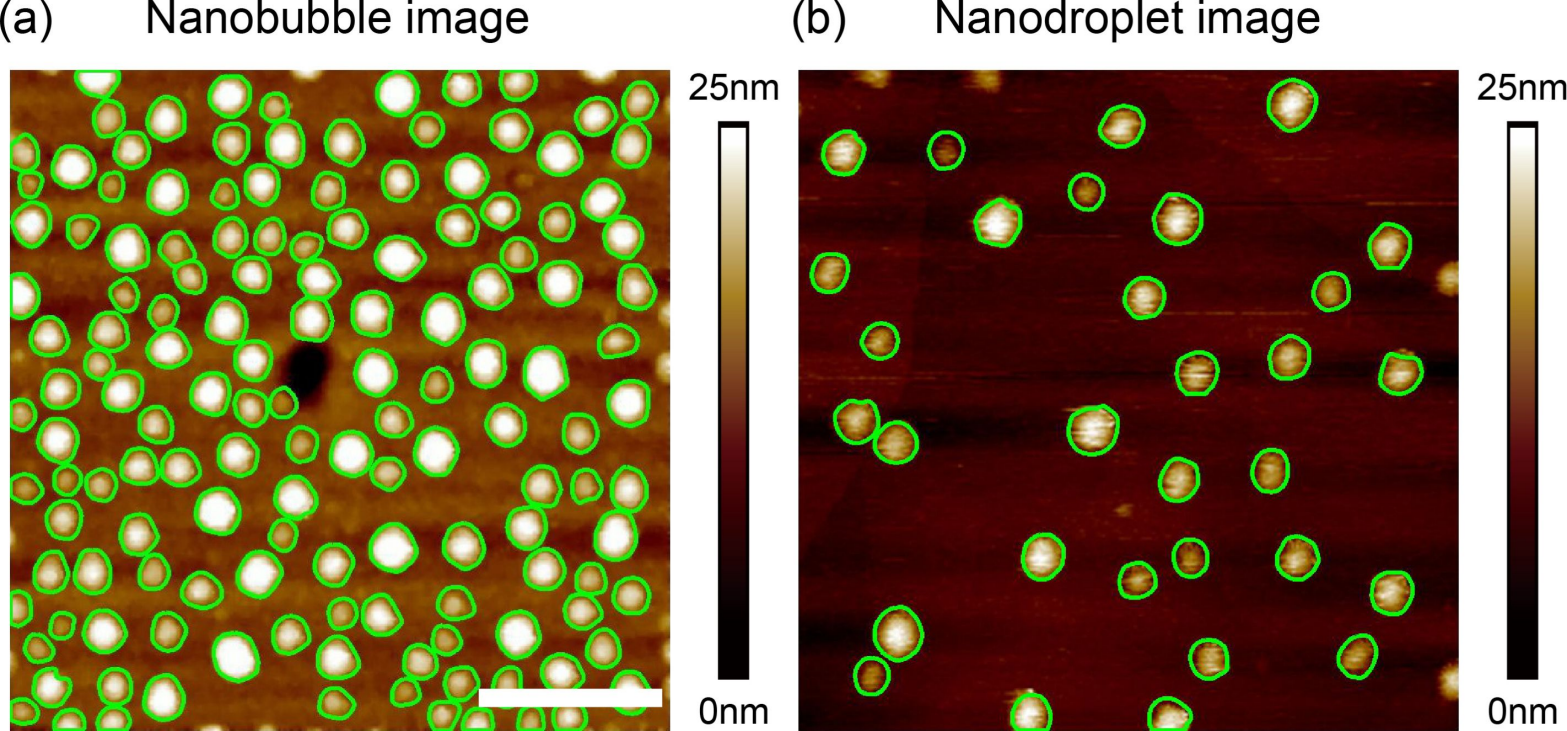
Segmentation result for a NB (a) and ND (b) in AFM images using the proposed method. The scale bar is 500 nm.

NBs and NDs are both spherical-cap-shaped objects. Once they are segmented, the morphological characterization can be automatically conducted for AFM images. Figure 10a and Figure 10b show the segmentation results for a NB and ND in the AFM image, respectively.

**Figure 11:**
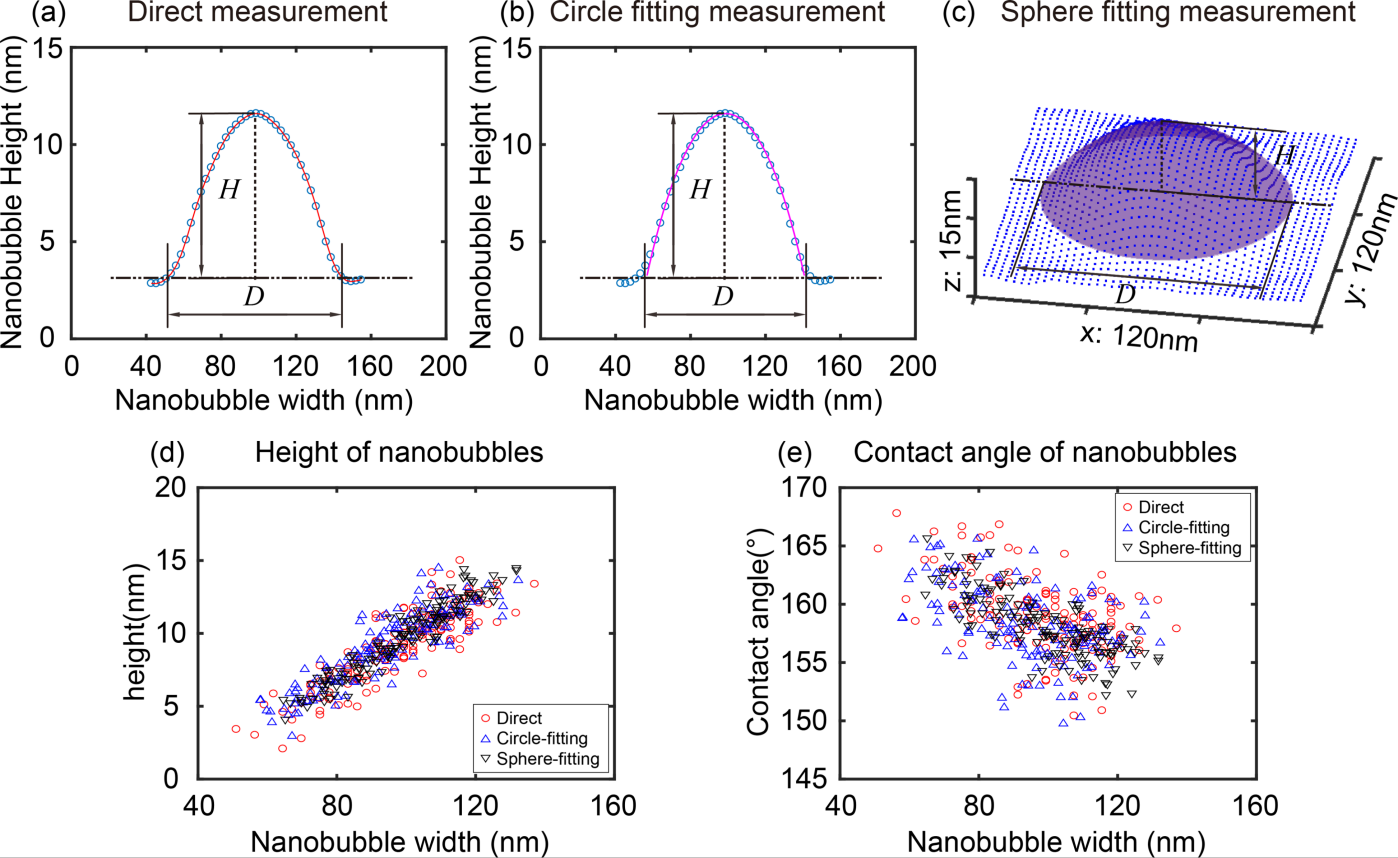
Comparison of three different automated morphological characterization methods for nanobubbles. (a), (b) and (c) are the demonstration of the direct measurement, circle fitting, and sphere fitting methods, respectively. (d) and (e) are the comparison of the height and contact angle of nanobubbles obtained with the three methods for the AFM image shown in Figure 10a.

After segmentation, the morphological characterization of NBs and NDs can be implemented using three approaches, as shown in Figure 11. Here they are referred to as direct measurement, circle fitting, and sphere fitting methods. The direct measurement method is applied to cross sections of NBs and NDs, as shown in Figure 11a. After cross sections are extracted, the height *H* and width *D* can be directly measured. The radius of curvature *R* and contact angle θ can then be obtained with *H* and *D*.

The circle fitting method also applies to cross sections. In the method, the data points on NBs and NDs are fitted as circles using the least squares method, as shown in Figure 11b. From the fitted parameters, the parameters (including radius of curvature) of the circle can be obtained. The width *D* and height *H* can be obtained with the determined base line height from segmentation. The sphere fitting method fits all data points on the segmented NB/ND surfaces as spheres (Figure 11c). After fitting, the parameters of the spheres (including radius of curvature) can be directly obtained. Then the height, *H*, and width, *D*, can be obtained with the determined base line height around NB/ND boundaries

In the automated morphological characterization of NBs and NDs, their centers and base line heights need to be automatically detected. In this study, the centers were extracted with the centroid method within the segmented areas [27]. The average height value of the detected boundaries is taken as the height of base lines and used to extract data points on NB/ND surfaces and determine their *H* and *D*.

Here we take height and contact angle of NBs as examples for the comparison of the three methods. Figure 11d and Figure 11e show the measurement result of the height and the contact angle from the direct measurement, circle fitting, and sphere fitting methods, respectively. Statistically, the three methods give close measurement results, especially for the direct measurement and circle fitting methods. The sphere fitting method gives a more convergent distribution of the contact angle. This is because the sphere fitting measurement uses more data points compared with the other two methods.

**Figure 12:**
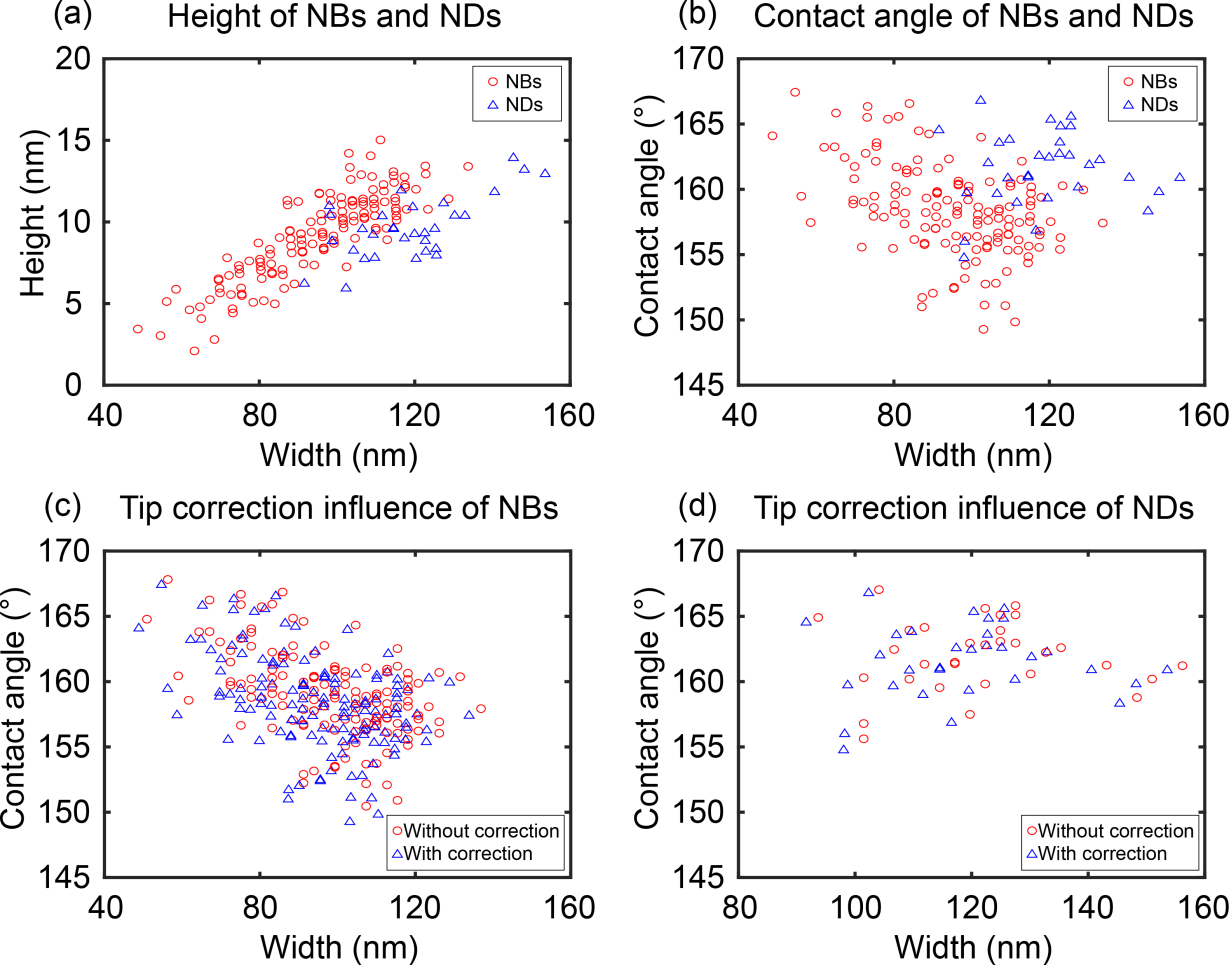
Automated morphological characterization of nanobubbles (NBs) and nanodroplets (NDs). (a) The height as a function of width for NBs and NDs after tip correction in AFM images shown in Figure 10. (b) The contact angle as a function of width for the NBs and NDs. The contact angle decreases with increasing NB/ND width. Comparison of the contact angle and width of NBs (c) and NDs (d) before and after tip correction. One can see that the limited tip radius causes an overestimation of the NB/ND width and contact angle.

As mentioned earlier, due to the finite size of AFM tips, the AFM images are actually the convolution of AFM tips with the real topography of samples. Here NB/ND characterization was implemented after tip correction (see Equations 1–3). Figure 12a and Figure 12b show the result of the automated measurement of the height and contact angle as a function of the width for NBs and NDs shown in Figure 10, respectively. One can see that the NB/ND height increases with increasing width. However, the contact angle decreases with increasing width from 170° to 150°. This result is consistent with those previously reported [17,23,32]. Our study also reveals that the contact angle of NDs is slightly higher than that of NBs, which is consistent with the results presented elsewhere [37–38]. The influence of the limited tip radius on the contact angle is also studied, as shown in Figure 12c and Figure 12d. One can see that the cantilever tip radius causes an overestimation of the NB/ND width and contact angle. The width error introduced by the applied tip radius is about 3%, while the contact angle error is near 0.4%.

## Conclusion

Current automated NB/ND segmentation methods suffer from the uneven background in AFM images and inaccurate boundary detection. In this study, we have developed a two-step approach to segment NBs and NDs in AFM images to obtain robust and optimized segmentation results. The spherical Hough transform was first used to preliminarily detect NBs and NDs. The contour expansion operation was then applied to obtain the optimized boundary detection. The result shows the proposed method can achieve an improved performance compared to the thresholding and CHT methods. All NBs were correctly detected in AFM images. Moreover, by comparing the NB segmentation result for AFM images with/without flattening, the proposed method shows strong robustness in processing AFM images with an uneven background. Following the successful segmentation, the morphological characterization of NBs and NDs was implemented. Three methods (direct measurement, circle fitting, and sphere fitting) were applied to automatically extract their height, width, and contact angle. The results show that the three methods statistically provide a close estimation of their morphological properties. We believe the proposed method provides a valid, useful tool in NB/ND related studies. Additionally, it is useful for the general segmentation of images containing spherical objects and those requiring accurate boundary detection in many other applications.
